# Evaluation of ^64^Cu-Labeled New Anti-EGFR Antibody NCAB001 with Intraperitoneal Injection for Early PET Diagnosis of Pancreatic Cancer in Orthotopic Tumor-Xenografted Mice and Nonhuman Primates

**DOI:** 10.3390/ph14100950

**Published:** 2021-09-23

**Authors:** Hiroki Matsumoto, Tadashi Watabe, Chika Igarashi, Tomoko Tachibana, Fukiko Hihara, Atsuo Waki, Ming-Rong Zhang, Hideaki Tashima, Taiga Yamaya, Kazuhiro Ooe, Eku Shimosegawa, Jun Hatazawa, Sei Yoshida, Kenichiro Naito, Hiroaki Kurihara, Makoto Ueno, Kimiteru Ito, Tatsuya Higashi, Yukie Yoshii

**Affiliations:** 1 Institute for Quantum Medical Science, National Institutes for Quantum and Radiological Science and Technology, Chiba 263-8555, Japan; matsumoto.hiroki2@qst.go.jp (H.M.); igarashi.chika@qst.go.jp (C.I.); tachibana.tomoko@qst.go.jp (T.T.); hihara.fukiko@qst.go.jp (F.H.); waki.atsuo@qst.go.jp (A.W.); zhang.ming-rong@qst.go.jp (M.-R.Z.); tashima.hideaki@qst.go.jp (H.T.); yamaya.taiga@qst.go.jp (T.Y.); higashi.tatsuya@qst.go.jp (T.H.); 2 Department of Diagnostic Radiology, Kanagawa Cancer Center, Kanagawa 241-8515, Japan; h-kurihara@kcch.jp; 3 Department of Nuclear Medicine and Tracer Kinetics, Osaka University Graduate School of Medicine, Osaka 565-0871, Japan; watabe@tracer.med.osaka-u.ac.jp (T.W.); ooe@tracer.med.osaka-u.ac.jp (K.O.); eku@mi.med.osaka-u.ac.jp (E.S.); hatazawa-jun@jrias.or.jp (J.H.); 4 Department of Research, NanoCarrier Co., Ltd., Tokyo 104-0031, Japan; yoshida@nanocarrier.co.jp (S.Y.); naito@nanocarrier.co.jp (K.N.); 5 Department of Gastroenterology, Kanagawa Cancer Center, Kanagawa 241-8515, Japan; uenom@kcch.jp; 6 Department of Diagnostic Radiology, National Cancer Center Hospital, Tokyo 104-0045, Japan; kimito@ncc.go.jp

**Keywords:** ^64^Cu-NCAB001, ipPET, radiation dosimetry, nonhuman primate, preclinical safety

## Abstract

Objectives: To improve the prognosis of pancreatic cancer, new imaging methods to identify tumor lesions at a size of <1 cm are urgently needed. To approach this clinical issue, we developed a new method to detect small tumor lesions in the pancreas (≥3 mm) by positron emission tomography (PET) using an intraperitoneally (ip)-administered ^64^Cu-labeled new anti-epidermal growth factor receptor (EGFR) antibody (encoded as NCAB001), called ^64^Cu-NCAB001 ipPET. Methods: NCAB001 was manufactured under cGMP conditions and labeled with ^64^Cu. The radiochemical and biological properties of ^64^Cu-NCAB001 were evaluated. Tumor uptake of an ip-administered ^64^Cu-NCAB001 in mice with orthotopic pancreatic tumor xPA1-DC xenografts was also evaluated. Pharmacokinetics and radiation dosimetry were examined using PET images acquired after the ip administration of ^64^Cu-NCAB001 into cynomolgus monkeys with pharmacologic safety monitoring. Results: Radio-chromatography, cell-binding assays, and biodistribution of ^64^Cu-NCAB001 in mice were identical to those of our previous data with clinically available cetuximab. Small tumor lesions in the pancreas (≥3 mm) of mice could be identified by ^64^Cu-NCAB001 ipPET. The ip administration of ^64^Cu-NCAB001 into monkeys was safely conducted using ultrasound imaging. PET images in monkeys showed that ip-administered ^64^Cu-NCAB001 was distributed throughout the intraperitoneal cavity for up to 6 h and cleared thereafter. Most of the radioactivity was distributed in the liver and the large intestine. The radioactivity around the pancreas became negligible 24 h after administration. The estimated human effective dose was 0.0174 mSv/MBq. Conclusion: Our data support the initiation of clinical trials of ^64^Cu-NCAB001 ipPET to transfer this promising tool for the early diagnosis of pancreatic cancers.

## 1. Introduction

Early diagnosis and treatment of pancreatic cancer is a clinical challenge, and the 5-year overall survival rate is <10% [1,2,3,4]. Previous clinical studies have reported that patient survival could be significantly improved by surgery if the tumor could be detected at a resectable size (<1 cm) [5,6,7]. To identify the tumor lesions in the pancreas earlier, several new plasma biomarkers, such as proteins, miRNAs, and metabolites, are under clinical development globally [8,9,10,11,12,13]. However, imaging diagnosis for the localization of pancreatic cancer at a size of <1 cm using current modalities remains challenging. The epidermal growth factor receptor (EGFR) is a good target for PET imaging of early pancreatic cancer, as its overexpression is observed in about 90% of pancreatic cancer cases [14,15]. Moreover, intraoperative multi-instrument fluorescence imaging using the anti-EGFR antibody cetuximab conjugated to IRDye800 could detect pancreatic cancer in patients with a sensitivity of 96.1% [16]. We previously labeled clinically available cetuximab (Erbitux) with ^64^Cu and intraperitoneally (ip) administered it to mice for PET imaging and therapy [17,18,19]. In mice with orthotopic human pancreatic tumor xPA1-DC xenografts, tumor lesions ≥3 mm were clearly detected in the pancreas using this approach [19]. In contrast, we could not detect lesions ≥3 mm in diameter after intravenous (iv) administration of ^64^Cu-cetuximab or iv/ip administration of ^18^F-fluorodeoxyglucose (^18^F-FDG). For the translation of this approach into clinical practice, the efficient and sustainable production of antibody is needed. Cetuximab has been manufactured using murine hybridoma cells (Sp2/O-Ag14) [20]. In this study, we used Chinese hamster ovary (CHO) cells instead, as the yield in this cell type is higher than that in Sp2/O cells [21]; we established the manufacturing process for a new anti-EGFR monoclonal antibody encoded as NCAB001 (with the same amino acid sequence as cetuximab) in CHO cells. We labeled NCAB001 with ^64^Cu and evaluated the feasibility of using ^64^Cu-NCAB001 by radio-thin-layer chromatography (radio-TLC), cell-binding assays, PET studies using mice with small orthotopic pancreatic tumor xPA1-DC xenografts, and biodistribution in mice. We obtained identical results using ^64^Cu-NCAB001 and ^64^Cu-cetuximab, and decided to initiate subsequent preclinical studies of ^64^Cu-NCAB001 in accordance with the regulatory guidelines for an investigational new drug application of imaging radiopharmaceuticals [22]. Accordingly, we evaluated the biodistribution, radiation dosimetry, and pharmacologic safety of ip-administrated ^64^Cu-NCAB001 in non-human primates. Cynomolgus monkeys (*Macaca fascicularis*) were chosen for the following reasons: (i) cetuximab is a mouse–human chimeric antibody targeting human EGFR; (ii) cynomolgus monkeys are genetically, anatomically, and physiologically similar to humans; and (iii) cynomolgus monkeys have been used for the preclinical safety assessment of cetuximab (Erbitux) [20].

Here, we report the efficient and sustainable production of a new anti-EGFR monoclonal antibody NCAB001, and the results of PET studies in mice with orthotopic tumors and non-human primates without tumors. We also report on the biodistribution, radiation dosimetry, and pharmacologic safety of ^64^Cu-NCAB001, and present radiation dose estimates for future clinical studies of ipPET.

## 2. Results

### 2.1. Radiochemical Purity and Cell-Binding Property Evaluation

**Figure 1:**
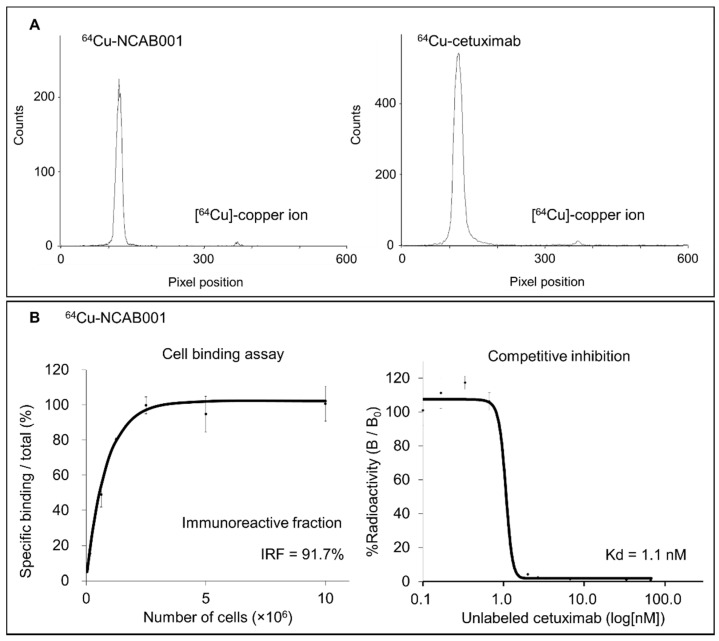
Radiochemical and biological evaluation of ^64^Cu-NCAB001. (**A**) Representative thin-layer chromatography (TLC) chromatogram of ^64^Cu-NCAB001 and ^64^Cu-cetuximab. (**B**) Cell-binding assay of ^64^Cu-NCAB001. The immunoreactive fraction (IRF) and the dissociation constant (Kd) of ^64^Cu-NCAB001 were determined to be 91.7% and 1.1 nM, respectively.

The anti-EGFR antibody NCAB001 was manufactured under current good manufacturing practice (cGMP), and quality tests were performed (Supplementary Table S1). Radiolabeling of NCAB001 and cetuximab was performed, and the representative radio-TLC chromatograms of ^64^Cu-NCAB001 and ^64^Cu-cetuximab immediately after radiolabeling were shown in Figure 1A. The retention factors of ^64^Cu-NCAB001 and ^64^Cu-cetuximab on TLC were 0–0.1; these were identical to that of the reference standard cetuximab. The retention factors of the radiochemical impurities of ^64^Cu-NCAB001 and ^64^Cu-cetuximab on TLC were identical to that of the free [^64^Cu] copper ion (0.8). The radiochemical purities of ^64^Cu-NCAB001 and ^64^Cu-cetuximab were ≥95%.

The cell-binding assay using ^64^Cu-NCAB001 (Figure 1B) revealed that the immunoreactive fraction (IRF) and dissociation constant (Kd) of ^64^Cu-NCAB001 were 91.7% and 1.1 nM, respectively. We have previously reported that the IRF and Kd of ^64^Cu-cetuximab were 96.4% and 1.0 nM, respectively [17]. These results indicated that ^64^Cu-NCAB001 and ^64^Cu-cetuximab were radiochemically and biochemically identical.

### 2.2. ipPET Imaging and Biodistribution in Mice

**Figure 2:**
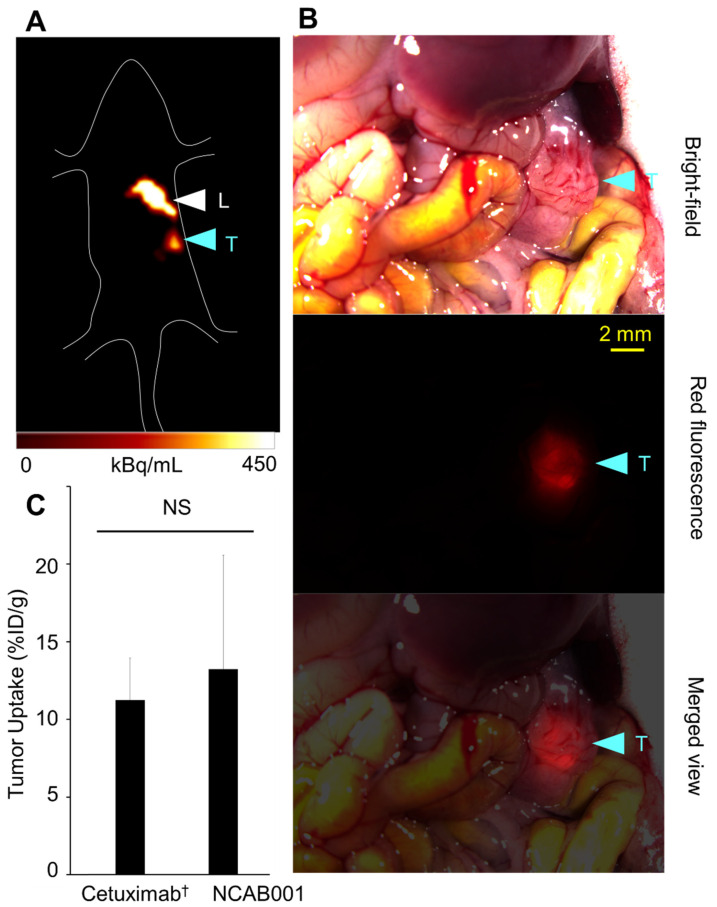
Representative images of the mouse model of an orthotopic xPA1-DC pancreatic tumor xenograft. (**A**) Representative image (coronal view) of ^64^Cu-NCAB001 intraperitoneal positron emission tomography (ipPET) obtained 24 h after the administration. Tumor and liver are shown using blue and white arrowheads, respectively. L = liver, T = tumor. (**B**) Overview and isolated pancreas with tumor observed using a stereoscopic fluorescence microscope (bright-field, red fluorescence, and merged views). The xPA1-DC tumor (3 mm × 3 mm) was identified at the site detected by PET. (**C**) Tumor uptake of ^64^Cu-NCAB001 measured after PET imaging. NS: no significant differences in tumor uptake between cetuximab and NCAB001. ^†^ Cetuximab data are from our previous reports [17].

**Figure 3:**
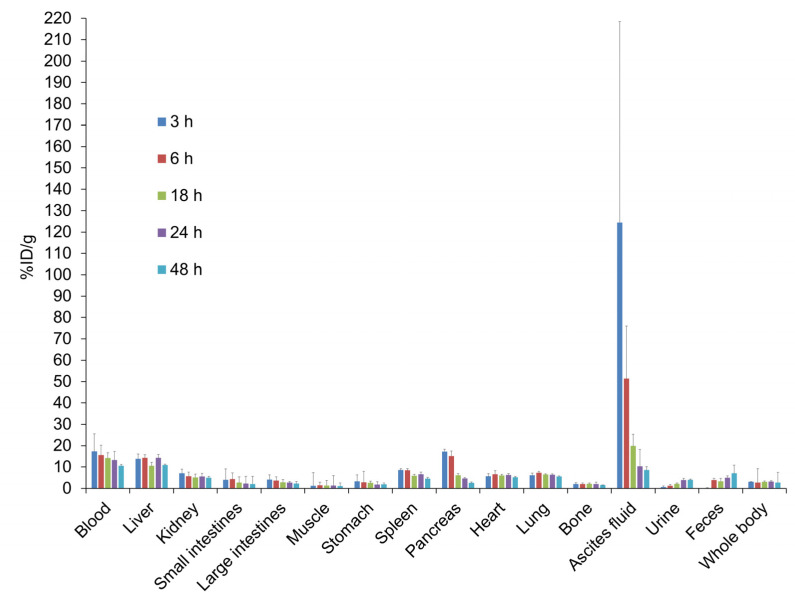
Biodistribution of the intraperitoneally administrated ^64^Cu-NCAB001 in mice (*n* = 4). Data are shown as the %injected dose (ID)/g for the organs, blood, and ascites fluid, and the %ID for the urine and feces.

Figure 2 shows a representative PET image and tumor uptake (%ID/g) of ip-administered ^64^Cu-NCAB001 in mice with an orthotopic xPA1-DC pancreatic tumor xenograft. A tumor sized 3 mm × 3 mm located inside the pancreas was clearly identified by PET. We obtained the same result with mice carrying a deeply located intraperitoneal colon tumor xenograft (Supplementary Figure S1), suggesting that tumors located inside the peritoneal cavity can be identified by ^64^Cu-NCAB001 ipPET. The organ distribution of ^64^Cu-NCAB001 was evaluated in non-tumor-bearing mice (Figure 3), and the human absorption dose was estimated (Supplementary Table S2). The results of ^64^Cu- NCAB001 in this study were identical to our previous results obtained using ^64^Cu-cetuximab [17,18,19].

### 2.3. ipPET Imaging, Absorbed Radiation Dose Estimations, and Pharmacological Safety Assessments in Monkeys

**Figure 4:**
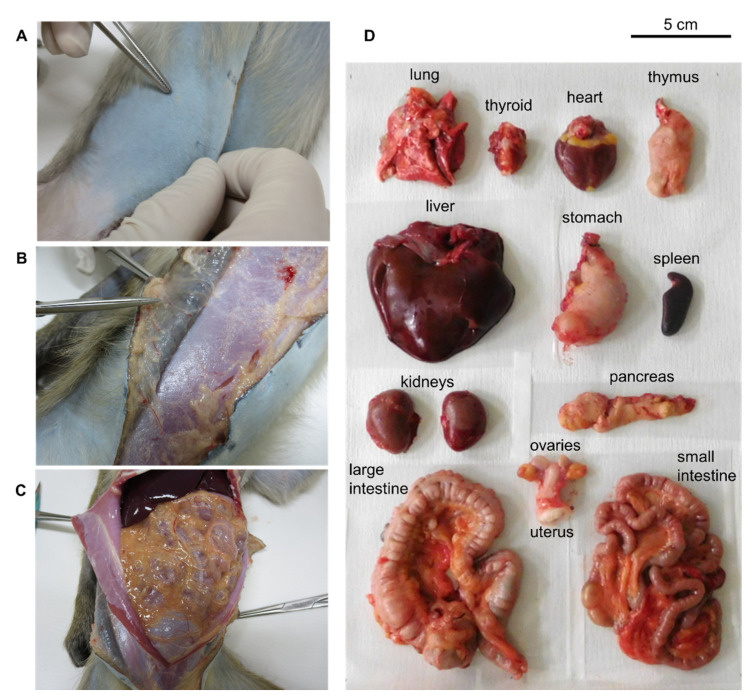
Local irritation and autopsy examination of animals after the intraperitoneal administration of ^64^Cu-NCAB001. (**A**) Surface of the abdominal skin, (**B**) surface of the abdominal muscle, and (**C**) outer surface of the peritoneum containing the administration site. (**D**) Organs excised from the body. Administration-related abnormalities were not found in any organs.

The characteristics of the experimental monkeys and the injected ^64^Cu-NCAB001 doses are listed in Table 1. Ultrasound imaging allowed for the safe intraperitoneal administration of ^64^Cu-NCAB001 to monkeys (Supplementary Video S1). All the monitored clinical parameters were within the reference ranges throughout the experiments (Supplementary Figure S2). No adverse events were noted during or after the PET scanning period. Upon sacrifice, an autopsy was performed, especially at the injection site and organs in the intraperitoneal cavity (Figure 4). Administration-related changes suggesting local irritation or toxicity such as intestinal inflammation were not observed.

**Figure 5:**
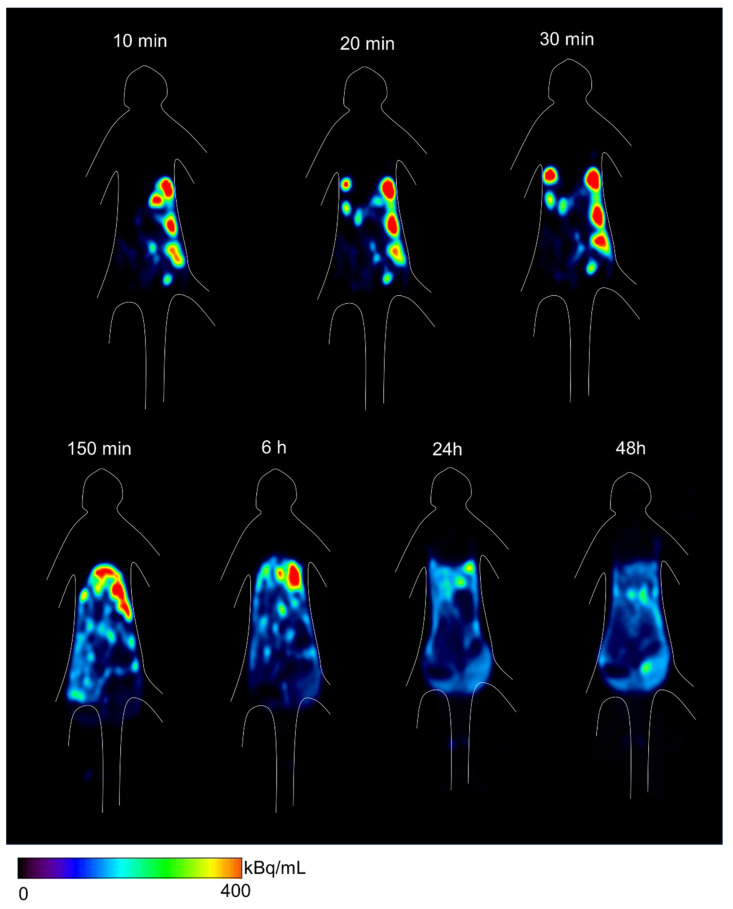
Positron emission tomography (PET) images of distribution of ^64^Cu-NCAB001 radioactivity in a representative monkey at different time points after intraperitoneal administration. Representative PET images with coronal view containing pancreas.

**Figure 6:**
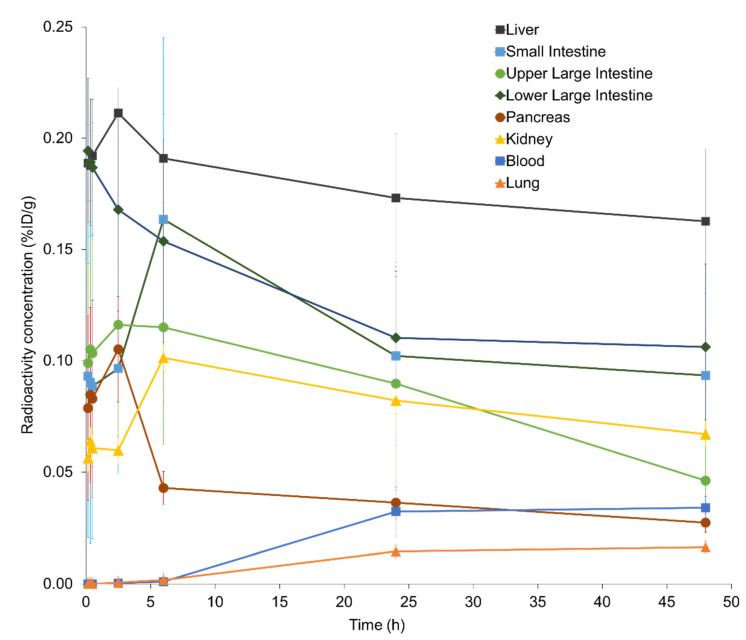
Time–activity curves of intraperitoneally administrated ^64^Cu-NCAB001 in key organs of monkeys. Data are shown as the %injected dose (ID)/g for each organ.

PET images revealed that ip-administered ^64^Cu-NCAB001 was distributed throughout the intraperitoneal cavity for up to 6 h and cleared thereafter (Figure 5). The radioactivity around the pancreas was negligible 24 h after administration. Individual time–activity curves of the key organs for ^64^Cu-NCAB001-derived radioactivity are shown in Figure 6. The peak of the total radioactivity concentration in each organ was observed at 2.5–6 h after ip administration.

The estimated human radiation-absorbed doses of the individual organ per unit administered activity of ^64^Cu-NCAB001 are presented in Table 2. The liver (0.107 mSv/MBq) and other organs in the peritoneal cavity (0.03–0.09 mSv/MBq) received relatively high doses, but these doses were sufficiently below the reported radiation tolerance dose (Supplementary Table S3) [23]. The effective dose was estimated to be 0.0174 mSv/MBq.

## 3. Discussion

In this study, we evaluated a new approach to detect small tumors (≥3 mm), called ^64^Cu-NCAB001 ipPET, in mice with orthotopic pancreatic tumor xenografts. We also evaluated the biodistribution, radiation dosimetry, and safety pharmacology of ^64^Cu-NCAB001 ipPET in monkeys.

Our previous studies described the benefits of ip-administrated ^64^Cu-cetuximab for the detection of early pancreatic cancers in mouse models using cetuximab (Erbitux) as a starting material for this radiotracer [17,18,19]. To secure a sustainable and efficient supply of the material for this new radiotracer, we established the manufacturing process of the new anti-EGFR monoclonal antibody coded NCAB001. The results of ^64^Cu-NCAB001 representing its radiochemical purity and cell-binding properties (Figure 1), detection of small and orthotopic pancreatic tumors by PET imaging (Figure 2), and biodistribution and dosimetry (Figure 3 and Supplementary Table S2) were identical to those of our previous reports using ^64^Cu-cetuximab [17,18,19]. Therefore, we decided to use ^64^Cu-NCAB001 for further preclinical studies, including the monkey studies reported herein, to initiate the first-in-human imaging studies of this agent.

The ip administration of ^64^Cu-NCAB001 was safely conducted in monkeys with the aid of ultrasound imaging. In this study, the total amount of non-radiolabeled PCTA-NCAB001 administered to each monkey was 100 μg, which is approximately 7000-fold less than the initial single dose of cetuximab (400 mg/m^2^, iv drip infusion) [20]. This finding supports our plan to develop an investigational formulation of ^64^Cu-NCAB001 with the total amount of non-radiolabeled PCTA-NCAB001 at less than 100 μg/dose.

PET images in monkeys showed that ip-administered ^64^Cu-NCAB001 was distributed throughout the intraperitoneal cavity for up to 6 h and cleared thereafter (Figure 5). The radioactivity around the pancreas was negligible 24 h after administration. This is one of the key findings in this study, as the background activities around pancreatic cancer can be expected to be negligible in the PET images of patients in future clinical studies. Most of the radioactivity was distributed in the liver and large intestine, with the peak observed at 2.5–6 h after ip administration. The liver (0.107 mSv/MBq) and other organs in the peritoneal cavity (0.03–0.09 mSv/MBq) received relatively high doses (Table 2). The estimated effective dose was 0.0174 mSv/MBq. A previous study reported that 130 MBq of ^64^Cu-DOTA-trastuzumab could be used for the identification of HER2-positive tumors by PET in primary and metastatic breast cancer patients [24]. Considering the same dose as ^64^Cu-DOTA-trastuzumab for ^64^Cu-NCAB001 ipPET, 130 MBq of ^64^Cu-NCAB001 is estimated to be 1000 times lower than the reported radiation tolerance dose (Supplementary Table S3) [23]. The effective dose of 130 MBq ^64^Cu-NCAB001 ipPET is estimated to be equivalent to the dose caused by current clinical PET procedures, such as ^18^F-FDG PET [25]. Based on our estimates, 130 MBq of ^64^Cu-NCAB001 per injection should be an acceptable starting dose for first-in-human clinical PET studies in patients with early pancreatic cancer.

This study has some limitations. The feasibility of using ip-administrated ^64^Cu-NCAB001 to detect small tumors in the pancreas should be investigated in future clinical studies with patients. In addition, the procedures for the intraperitoneal administration of ^64^Cu-NCAB001 with ultrasound imaging will have to be carefully established in our planned clinical trials.

## 4. Materials and Methods

### 4.1. Cell Culture and Experimental Animals

CHO cells were obtained from Thermo Fisher Scientific (FreeStyle MAX Expression System and CHO-S Cells) and used for the production of NCAB001. Human colon cancer HCT116 cells were obtained from the American Type Cell Collection (CCL-247, RRID: CVCL_0291) and cultured for cell-binding assays, as previously reported [17]. Human pancreatic tumor xPA1-DC cells expressing red fluorescent protein were obtained from AntiCancer and cultured for imaging studies in mice, as we previously reported [18,19].

Female BALB/c nude mice (six-week-old, 15–20 g body weight) were obtained from Japan SLC (Hamamatsu, Japan). Four cynomolgus monkeys (two male and two female monkeys; body weight approximately 2.5 kg) were obtained from Hamri (Koga, Japan). The experiments in this study were performed in accordance with the laws and guidelines for animal welfare as per the Japanese Government and the National Research Council. All animal experimental procedures were approved by the Animal Ethics Committee of our institutions and conducted in compliance with our institutional guidelines. Animals were housed in individual cages with appropriate bedding, and a 12 h light/dark cycle was applied. For the mice, solid food and municipal tap water were provided without restriction. For the monkeys, solid food was provided once daily in the morning, supplementary fruits once daily in the evening, and municipal tap water ad libitum. The animals were weighed and used for the experimental studies after >7 days of quarantine.

### 4.2. Manufacturing and Quality Control of NCAB001

The master cell banks for NCAB001 and NCAB001 were manufactured under cGMP conditions at Mycenax Biotech Inc. (Jhunan, Taiwan). Briefly, a DNA strand coding cetuximab was transfected into CHO cells using a standard procedure [26]. After cell clarification, monoclonal antibodies were captured by r-protein A affinity chromatography (Toyopearl AF-rProtein A HC-650F, Tosoh Bioscience, Tokyo, Japan), followed by low-pH treatment for virus inactivation, polishing purification by mixed-mode chromatography (Toyopearl Mx-Trp-650M, Tosoh Bioscience), and viral filtration. The final NCAB001 product was formulated with phosphate buffer, and quality tests were performed (Supplementary Table S1).

### 4.3. Radiolabeling and Cell-Binding Assay

^64^Cu labeling of NCAB001 was conducted as reported previously [27,28,29] with some modifications. The bifunctional chelator 3,6,9,15-tetraazabicyclo[9.3.1] pentadeca-1(15),11,13-triene-4-S-(4-isothiocyanatobenzyl)-3,6,9-triacetic acid (p-SCN-Bn-PCTA, Macrocyclics, Plano, TX, USA) was used. ^64^Cu was produced using a cyclotron and purified according to previously reported procedures [30]. ^64^CuCl_2_ was dissolved in acetate buffer (0.1 M, pH 6.0), added to the PCTA-NCAB001 solution (2 mg/mL) at a 3:1 ratio (vol: vol), and incubated for 1 h at 40 °C. The radiochemical purity of ^64^Cu-NCAB001 was determined using radio-TLC. A silica gel 60 plate (Merck Millipore, Burlington, MA, USA) and a mobile phase comprising methanol and water (80:20 vol:vol) were used. The radioactivity on the TLC plates was quantitated using a bioimaging analyzer (FLA-7000, GE Healthcare Life Sciences, Marlborough, MA, USA), and the relative activity fraction of intact ^64^Cu-NCAB001 over the total radioactivity was calculated. Cetuximab was labeled with ^64^Cu, and the radiochemical purity of ^64^Cu-cetuximab was determined as described above.

Cell-binding assays using HCT116 cells were conducted, as we reported previously [17]. Briefly, cells were diluted in PBS with 1% bovine serum albumin (BSA) (Sigma-Aldrich, St. Louis, MO, USA) and incubated with ^64^Cu-NCAB001 on ice for 1 h. After washing, a γ-counter (1480 Automatic Gamma Counter Wizard 3; PerkinElmer, Waltham, MA, USA) was used to measure the radioactivity bound to the cells. The immunoreactivity of ^64^Cu-NCAB001 was determined using the Lindmo method [31]. For competitive inhibition assays, HCT116 cells were incubated with ^64^Cu-NCAB001 and various concentrations of unlabeled NCAB001 on ice for 1 h. After washing, the radioactivity bound to the cells was measured as described above. The dissociation constant was determined using the competitive inhibition assay data. These results were compared to those from our previous study using ^64^Cu-cetuximab [17].

### 4.4. Imaging and Biodistribution of ^64^Cu-NCAB001 in Mice

A mouse model of pancreatic cancer with an orthotopic xPA1-DC tumor xenograft was established as previously reported [19]. Briefly, xPA-1-DC cells (5 × 10^6^) in 25 μL of RPMI-1640 medium mixed with 25 μL of ice-cold extracellular matrix (Matrigel matrix, BD Biosciences, Franklin Lakes, NJ, USA) were injected into the pancreatic tail through the incision. One week later, the developed xPA-1-DC tumors were isolated and minced with a razor to obtain small (approximately 1 mm^3^) pieces. One tumor piece was slowly injected into the tail of the pancreas. Two weeks after tumor implantation, ^64^Cu-NCAB001 (7.4 MBq/mouse) was intraperitoneally administered to the mice (*n* = 6). PET images were obtained 24 h later with a human-sized OpenPET system developed by our group [32]. Immediately after PET imaging, tumors were isolated and weighed, and the radioactivity levels were measured using a γ-counter. The percentage of injected dose per gram (%ID/g) was evaluated as previously reported [18]. The organ distribution of ^64^Cu-NCAB001 was evaluated in non-tumor-bearing mice as previously reported [17]. These results were compared with our previous results with ^64^Cu-cetuximab [17].

### 4.5. ^64^Cu-NCAB001 ipPET Imaging in Monkeys

Before imaging, the monkeys were fasted overnight with unrestricted access to water. The animals were premedicated intramuscularly with ketamine (5 mg/kg, Daiichi Sankyo Propharma, Tokyo, Japan), xylazine hydrochloride (0.5 mg/kg, Bayer AG Leverkusen, Germany), and atropine sulfate (0.04 mg/kg, Mistubishi Tanabe Pharma, Osaka, Japan), followed by inhalation anesthesia with isoflurane (1–2%, MSD Animal Health, Tokyo, Japan), and oxygen delivered by a gas anesthesia system. The body temperature was maintained using a heating pad and hot water bags. A bedside monitor (Life Scope-P; BSM-4103, Nihon Kohden, Tokyo, Japan) was used to collect electrocardiogram, pulse oximetry, and respiration rate data throughout the imaging study. Subsequently, maropitant citrate (0.05 mg/kg, Zoetis Japan, Tokyo, Japan) was administered intramuscularly once daily to prevent vomiting. Four hours after recovery from anesthesia, the animals were fed solid food and some fruits.

**Figure 7:**
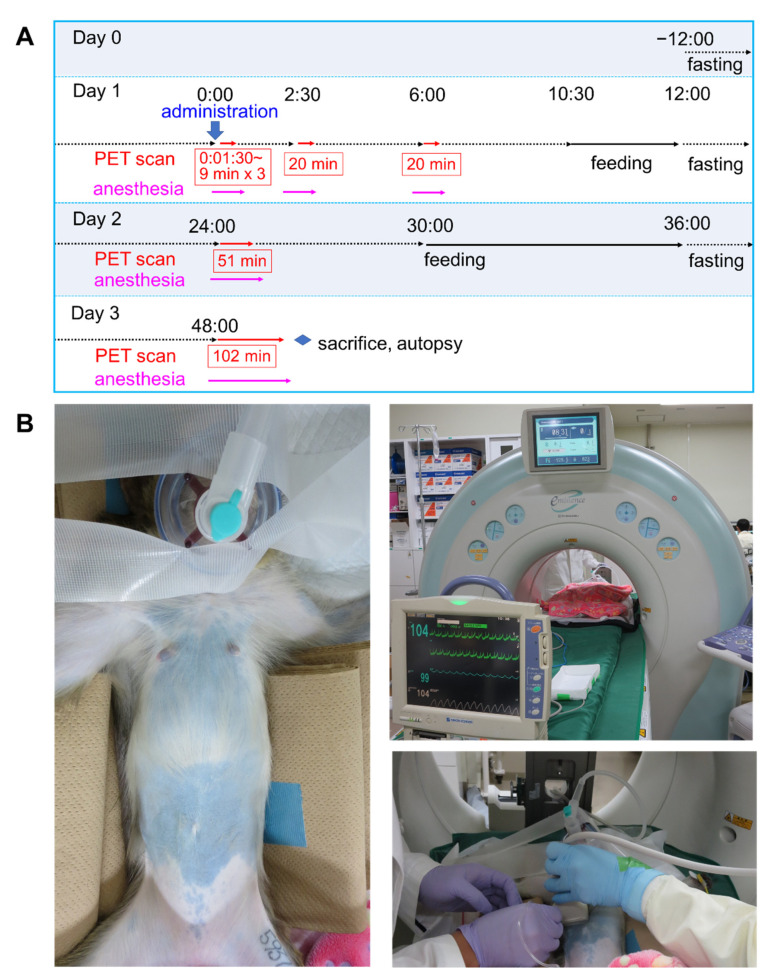
Time schedule and experimental setup of the monkey positron emission tomography (PET) scan. (**A**) Time schedule of the PET scans. Timing of the intraperitoneal (ip) administration is indicated as “0:00” of day 1. The PET scanning schedule is shown using red arrows and numbers. The anesthesia schedule is shown using pink arrows. (**B**) The experimental setup of a monkey PET scan. Experimental monkey under anesthesia (left), PET scanner and vital sign monitor (upper right), and ip administration route confirmation using an ultrasound scanner (lower right).

PET studies were performed using the Eminence-B (Shimadzu Corporation, Kyoto, Japan) PET system. The animals received a dose of 30 ± 0.9 MBq ^64^Cu-NCAB001 with a protein dose of 100 μg. Successful ip administration was confirmed using an ultrasound imaging system (Prosound α6, Hitachi Ltd., Tokyo, Japan) (Supplementary Video S1). The time schedule and experimental setup of the monkey PET scan are shown in Figure 7. The images were reconstructed with a dynamic row-action maximum likelihood algorithm with attenuation and scatter correction. With the assumption of a tissue density of 1 g/mL, the raw PET data were converted to kBq/mL using a cross-calibration factor predetermined between the dose calibrator (CRC-15R; Capintec, Florham Park, NJ, USA) and the PET instrument.

### 4.6. Image Analysis and Absorbed Radiation Dose Estimations from Monkey ipPET Images

Three-dimensional regions of interest in the PET images were manually delineated in the heart, liver, pancreas, small intestine, upper large intestine, lower large intestine, and kidney regions. The radioactivity concentration in each organ was expressed as %ID/g, and time–activity curves were obtained using PMOD (PMOD Technologies LLC). The %ID/g values of each organ were converted into the corresponding human values [33]. The human dosimetry of ^64^Cu-NCAB001 was then estimated using the reference adult male and female models in the OLINDA/EXM1.1 software [34].

### 4.7. Pharmacological Safety Assessment in Monkeys

The heart rates, respiration rates, pulse rates, electrocardiogram results, and pulse oximetry data of the experimental monkeys were monitored throughout the anesthesia procedure and PET imaging. Detailed physical examinations of the animals were conducted by skilled veterinarians and physicians closely before ^64^Cu-NCAB001 ipPET application and throughout the imaging session. Subsequently, the monkeys were sacrificed by intravenous (iv) administration of potassium chloride under ketamine and xylazine anesthesia. Autopsy, especially of the injection site and organs in the peritoneal cavity, was performed by experienced veterinarians and physicians.

### 4.8. Statistical Analysis

Data are expressed as means with standard deviations. *p* values were calculated using a 2-tailed *t*-test for comparisons between two groups. Statistical significance was set at *p* < 0.05.

## 5. Conclusions

The safety and feasibility of ^64^Cu-NCAB001 ipPET were demonstrated in a mouse model of small orthotopic pancreatic tumor xenografts and in non-human primates in this study. The radioactivity around the pancreas of the animals became negligible 24 h after administration, suggesting that low background activities around the pancreatic cancer can be expected in patients. Clinical studies of ^64^Cu-NCAB001 can be safely conducted to bring this new diagnostic option to future clinical practice.

## Figures and Tables

**Table 1:** Characteristics of the experimental monkeys, and the injected ^64^Cu-NCAB001 doses.

Animal ID	Sex	Age (Months)	Body Weight (kg)	Administered Activity (MBq)
5937366897	Female	26	2.5	31.0
4311432746	Female	22	2.7	31.1
9777568022	Male	27	2.5	29.3
8214867971	Male	27	2.5	31.3
All	--	25.5 ± 2.4	2.5 ± 0.1	30.7 ± 0.9

**Table 2:** Human absorbed doses of intraperitoneally administrated ^64^Cu-NCAB001: estimated from monkey biodistribution.

Target Organ	Total Estimated Absorbed Dose (mSv/MBq)						
	No. 1	No. 2	No. 3	No. 4	All		
Adrenals	6.76 × 10^−3^	7.36 × 10^−3^	3.75 × 10^−3^	5.03 × 10^−3^	5.73 × 10^−3^	±	1.65 × 10^−3^
Brain	4.11 × 10^−5^	4.11 × 10^−5^	1.89 × 10^−5^	2.97 × 10^−5^	3.27 × 10^−5^	±	1.07 × 10^−5^
Breasts	1.30 × 10^−3^	1.32 × 10^−3^	6.56 × 10^−4^	9.64 × 10^−4^	1.06 × 10^−3^	±	3.15 × 10^−4^
Gallbladder wall	1.29 × 10^−2^	1.47 × 10^−2^	8.52 × 10^−3^	1.09 × 10^−2^	1.18 × 10^−2^	±	2.66 × 10^−3^
Lower large intestinal wall	7.12 × 10^−2^	4.98 × 10^−2^	6.39 × 10^−2^	5.27 × 10^−2^	5.94 × 10^−2^	±	9.94 × 10^−3^
Small intestine	5.18 × 10^−2^	8.74 × 10^−2^	9.59 × 10^−2^	1.10 × 10^−1^	8.63 × 10^−2^	±	2.48 × 10^−2^
Stomach wall	3.72 × 10^−3^	4.43 × 10^−3^	3.01 × 10^−3^	3.51 × 10^−3^	3.67 × 10^−3^	±	5.89 × 10^−4^
Upper large intestinal wall	3.38 × 10^−2^	6.49 × 10^−2^	8.05 × 10^−2^	6.70 × 10^−2^	6.16 × 10^−2^	±	1.97 × 10^−2^
Heart wall	6.56 × 10^−3^	4.82 × 10^−3^	4.94 × 10^−3^	7.05 × 10^−3^	5.84 × 10^−3^	±	1.13 × 10^−3^
Kidneys	4.05 × 10^−2^	5.96 × 10^−2^	4.82 × 10^−2^	4.89 × 10^−2^	4.93 × 10^−2^	±	7.85 × 10^−3^
Liver	1.42 × 10^−1^	1.45 × 10^−1^	5.65 × 10^−2^	8.42 × 10^−2^	1.07 × 10^−1^	±	4.37 × 10^−2^
Lungs	7.35 × 10^−3^	5.43 × 10^−3^	2.12 × 10^−3^	6.34 × 10^−3^	5.31 × 10^−3^	±	2.27 × 10^−3^
Muscle	1.81 × 10^−3^	2.10 × 10^−3^	1.47 × 10^−3^	1.74 × 10^−3^	1.78 × 10^−3^	±	2.59 × 10^−4^
Ovaries	5.64 × 10^−3^	7.35 × 10^−3^			6.50 × 10^−3^	*	
Pancreas	3.37 × 10^−2^	3.56 × 10^−2^	2.71 × 10^−2^	3.00 × 10^−2^	3.16 × 10^−2^	±	3.80 × 10^−3^
Red marrow	2.27 × 10^−3^	2.71 × 10^−3^	2.19 × 10^−3^	2.53 × 10^−3^	2.43 × 10^−3^	±	2.39 × 10^−4^
Osteogenic cells	1.49 × 10^−3^	1.68 × 10^−3^	1.05 × 10^−3^	1.28 × 10^−3^	1.38 × 10^−3^	±	2.71 × 10^−4^
Skin	8.60 × 10^−4^	9.67 × 10^−4^	6.13 × 10^−4^	7.58 × 10^−4^	8.00 × 10^−4^	±	1.51 × 10^−4^
Spleen	2.49 × 10^−3^	3.03 × 10^−3^	1.95 × 10^−3^	2.25 × 10^−3^	2.43 × 10^−3^	±	4.57 × 10^−4^
Testes			4.42 × 10^−4^	4.37 × 10^−4^	4.40 × 10^−4^	*	
Thymus	1.18 × 10^−3^	1.12 × 10^−3^	6.22 × 10^−4^	9.31 × 10^−4^	9.63 × 10^−4^	±	2.51 × 10^−4^
Thyroid	2.62 × 10^−4^	2.58 × 10^−4^	1.11 × 10^−4^	1.74 × 10^−4^	2.01 × 10^−4^	±	7.26 × 10^−5^
Urinary bladder wall	1.66 × 10^−3^	2.05 × 10^−3^	1.92 × 10^−3^	1.97 × 10^−3^	1.90 × 10^−3^	±	1.69 × 10^−4^
Uterus	3.81 × 10^−3^	5.43 × 10^−3^			4.62 × 10^−3^	*	
Total body	6.29 × 10^−3^	7.10 × 10^−3^	4.34 × 10^−3^	5.43 × 10^−3^	5.79 × 10^−3^	±	1.18 × 10^−3^
Effective dose equivalent	2.32 × 10^−2^	2.75 × 10^−2^	2.32 × 10^−2^	2.49 × 10^−2^	2.47 × 10^−2^	±	2.03 × 10^−3^
Effective dose	1.94 × 10^−2^	1.77 × 10^−2^	1.55 × 10^−2^	1.69 × 10^−2^	1.74 × 10^−2^	±	1.63 × 10^−3^

* For reproductive organs, only the mean values are shown.

## Data Availability

Data are within the article and Supplementary Materials.

## Supplementary Materials

The following are available online at https://www.mdpi.com/article/10.3390/ph14100950/s1; Figure S1: Representative images of a mouse model with a deeply located intraperitoneal HCT116-RFP colorectal tumor xenograft, Figure S2: Vital sign monitoring during the intraperitoneal (ip) administration of ^64^Cu-NCAB001, Table S1: Batch release test results of NCAB001, Table S2: Human absorbed doses of intraperitoneally-administered ^64^Cu-NCAB001 estimated from mouse biodistribution, Table S3: Comparison between the estimated and tolerance doses in key organs during ^64^Cu-PCTA-cetuximab intraperitoneal positron emission tomography (ipPET), Video S1: The ultrasound imaging during the intraperitoneal administration of ^64^Cu-NCAB001 to a monkey.
