# Supplementary material for: Evaluation of 64Cu-Labeled New Anti-EGFR Antibody NCAB001 with Intraperitoneal Injection for Early PET Diagnosis of Pancreatic Cancer in Orthotopic Tumor-Xenografted Mice and Nonhuman Primates
Source: Pharmaceuticals (Basel). 2021 Sep 23;14(10):950. doi: 10.3390/ph14100950 (PMC8540406; doi:10.3390/ph14100950)
Supplement: Supplementary file 1 [file pharmaceuticals-14-00950-s001.zip › pharmaceuticals-1375349-supplementary.pdf]

Article

Evaluation of ^{64}Cu-labeled new anti-EGFR antibody NCAB001 with intraperitoneal injection for early PET diagnosis of pancreatic cancer in orthotopic tumor-xenografted mice and nonhuman primates

Hiroki Matsumoto, Tadashi Watabe, Chika Igarashi, Tomoko Tachibana, Fukiko Hihara, Atsuo Waki, Ming-Rong Zhang, Hideaki Tashima, Taiga Yamaya, Kazuhiro Ooe, Eku Shimosegawa, Jun Hatazawa, Sei Yoshida, Kenichiro Naito, Hiroaki Kurihara, Makoto Ueno, Kimiteru Ito, Tatsuya Higashi, and Yukie Yoshii

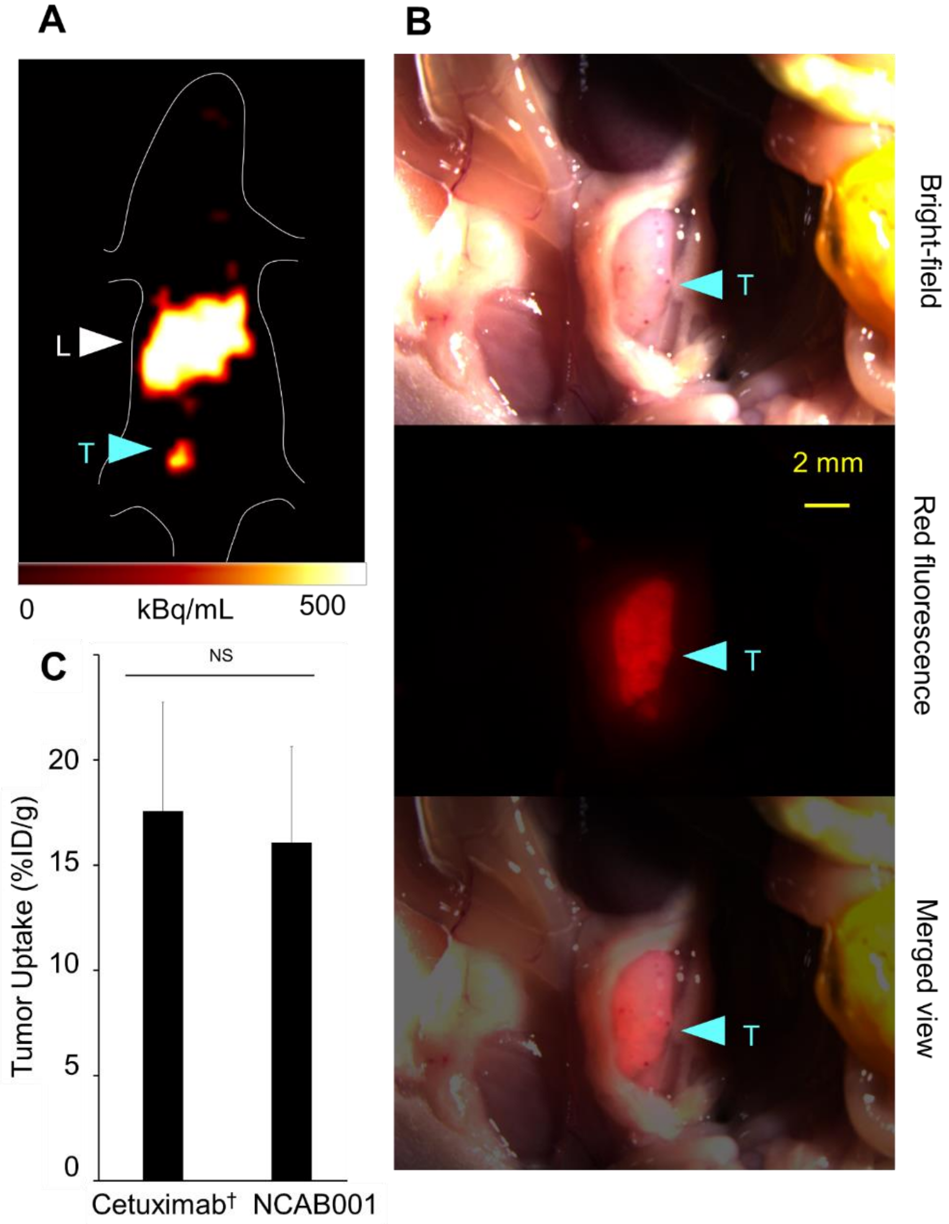

Supplementary Figure S1. Representative images of a mouse model with a deeply located intraperitoneal HCT116-RFP colorectal tumor xenograft.

(A) Representative image (coronal view) of ^{64}Cu-NCAB001 intraperitoneal positron emission tomography (ipPET). A mouse model with a deeply located intraperitoneal HCT116-RFP tumor xenograft was established as previously reported [17]. Briefly, HCT116-RFP cells (5 × 10^5) were suspended in 500 μL phosphate-buffered saline (PBS) and injected intraperitoneally to BALB/c nude mice. Two weeks after tumor implantation, ^{64}Cu-NCAB001 (7.4 MBq/mouse) was intraperitoneally administered to the mice (n = 4), and PET images were obtained 24 h later with a human-sized OpenPET system developed by our group [29]. The tumor and liver are indicated by blue and white arrowheads, respectively. L = liver, T = tumor. (B) Observations using a stereoscopic fluorescence microscope (bright field, red fluorescence, and merged views). The HCT116-RFP tumor (5 mm × 2 mm) was identified at the site detected by PET. (C) Tumor uptake of ^{64}Cu-cetuximab measured by PET imaging. Immediately after PET imaging, tumors were isolated, weighed, and radioactivity levels were measured using a γ-counter. The percentage of injected dose per gram (%ID/g) was evaluated as previously reported [18]. NS: no significant differences in tumor uptake between NCAB001 and cetuximab. † Cetuximab data have been taken from our previous report [17].

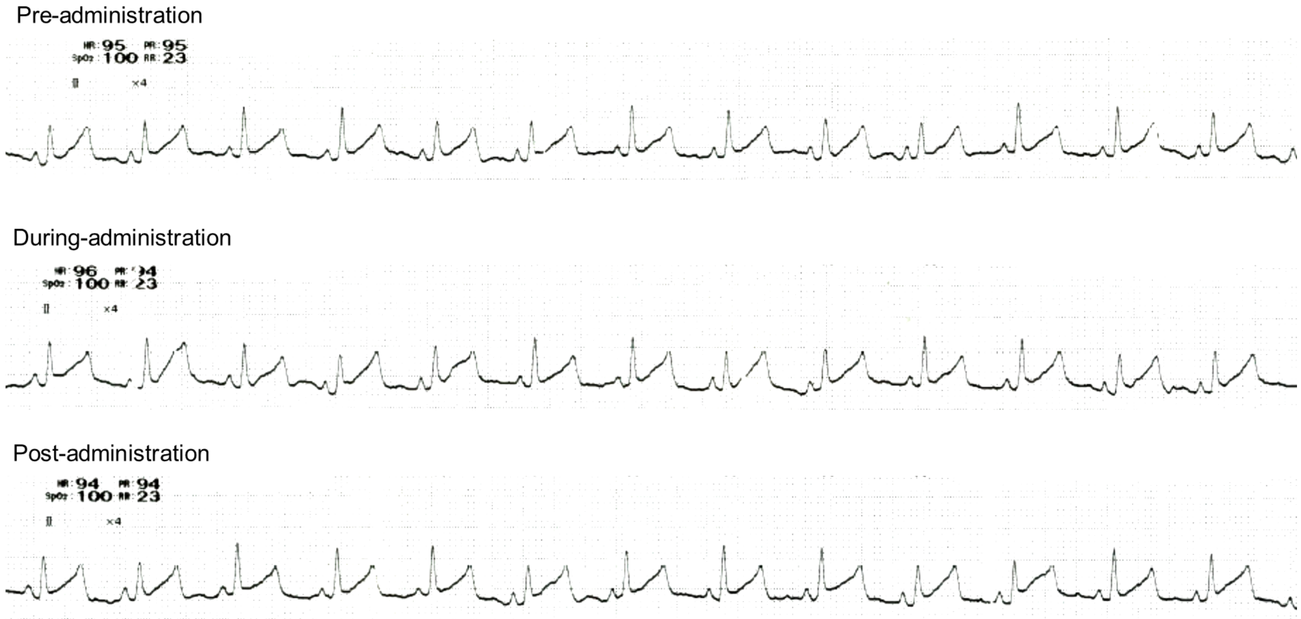

Supplementary Figure S2. Vital sign monitoring during the intraperitoneal (ip) administration of ^{64}Cu-NCAB001. Vital sign charts before (upper panel), during (middle panel), and after ip administration (lower panel) of ^{64}Cu-NCAB001.

Supplementary Video S1. Video shows the ultrasound imaging during the intraperitoneal administration of ^{64}Cu-NCAB001 to a monkey. The yellow arrowhead indicates the administration site inside the peritoneal cavity. White arrowheads indicate the stream of the administered ^{64}Cu-NCAB001 fluid inside the peritoneal cavity. See Supplementary_Video_S1.

Supplementary Table S1. Batch release test results of NCAB001

Type	Test	Methods	Results
**Potency**	Antigen binding activity	ELISA	93% of Erbitux
**Identity**	Isoform pattern	cIEF	Main peak: 8.0<br>pI range: 7.3 – 8.3
	Peptide mapping	RP-UPLC	The chromatogram corresponds to Erbitux
	N-Glycan profile	RP-UPLC	Total Glycan: 97.02 %<br>G0F-2GN: 0.14 %<br>G0-GN: 0.64 %<br>G0F-GN: 4.99 %<br>G0: 0.68 %<br>G0F: 80.27 %<br>Man-5: 2.20 %<br>G0FB+G1: 0.60 %<br>G1F: 4.58%<br>G1F′: 2.35%<br>G2F: 0.58 %
**Purity / impurity**	Charge variants by HPLC	IE-HPLC	Main peak: 52.44 %<br>Total acidic peak: 42.24 %<br>Total basic peak: 5.32 %
	CE-SDS	under reducing conditions	3.18% LMW
		under non-reducing conditions	1.42% NGHC
	Size variants	SE-HPLC	Purity: 98.86%<br>Impurity: 1%

ELISA: enzyme immunosorbent assay; cIEF: capillary isoelectric focusing; RP-UPLC: reverse phase ultra-performance liquid chromatography; IE-HPLC: ion-exchange high-performance liquid chromatography; CE-SDS: capillary electrophoresis sodium dodecyl sulfate; LMW: low molecular weight impurities; NGHC: non-glycosylated heavy chain; SE-HPLC: size-exclusion high-performance liquid chromatography.

Supplementary Table S2. Human absorbed doses of intraperitoneally-administered ^{64}Cu-NCAB001 estimated from mouse biodistribution

Target organ	Total estimated absorbed dose (mSv/MBq)
Adrenals	2.59×10^{-2}
Brain	2.67×10^{-4}
Breasts	2.98×10^{-3}
Gallbladder wall	4.14×10^{-3}
Lower large intestinal wall	2.12×10^{-2}
Small intestine	3.86×10^{-2}
Stomach wall	2.63×10^{-2}
Upper large intestinal wall	3.60×10^{-2}
Heart wall	6.94×10^{-2}
Kidneys	3.83×10^{-2}
Liver	4.63×10^{-2}
Lungs	7.04×10^{-3}
Muscle	1.01×10^{-2}
Ovaries	2.18×10^{-2}
Pancreas	9.67×10^{-2}
Red marrow	1.66×10^{-2}
Osteogenic cells	8.84×10^{-3}
Skin	2.33×10^{-3}
Spleen	2.80×10^{-2}
Testes	1.71×10^{-3}
Thymus	4.47×10^{-3}
Thyroid	1.24×10^{-3}
Urinary bladder wall	1.52×10^{-2}
Uterus	8.32×10^{-2}
Total body	1.03×10^{-2}
Effective Dose Equivalent	2.00×10^{-2}
Effective Dose	1.19×10^{-2}

Supplementary Table S3. Comparison between the estimated and tolerance doses in key organs during ^{64}Cu-PCTA-cetuximab intraperitoneal positron emission tomography (ipPET)

Organ*	Estimated dose in human (Sv/human)†	Tolerance dose of radiation (Sv)‡
Lower large intestinal wall	7.72×10^{-3}	35 – 50
Small intestine	1.12×10^{-2}	40
Upper large intestinal wall	8.00×10^{-3}	35 – 50
Kidneys	8.00×10^{-3}	23
Liver	6.41×10^{-3}	30
Pancreas	6.41×10^{-3}	30
Red marrow	3.15×10^{-4}	2.5

*Key organs in ^{64}Cu-PCTA-cetuximab ipPET are listed.

†Estimated doses with 130 MBq/human, which corresponds to the initial dose for the planned clinical trial of ^{64}Cu-PCTA-cetuximab ipPET.

‡Reported tolerated doses of radiation in organs [32]. The values were compared to the human absorbed doses of the ip-administrated ^{64}Cu-NCAB001 estimated from monkey biodistribution (Table 2 in the text).
